# Railway Track Inspection Using Deep Learning Based on Audio to Spectrogram Conversion: An on-the-Fly Approach

**DOI:** 10.3390/s22051983

**Published:** 2022-03-03

**Authors:** Muhammad Shadab Alam Hashmi, Muhammad Ibrahim, Imran Sarwar Bajwa, Hafeez-Ur-Rehman Siddiqui, Furqan Rustam, Ernesto Lee, Imran Ashraf

**Affiliations:** 1 Faculty of Computer Science and Information Technology, Khawaja Fareed University of Engineering and Information Technology, Rahim Yar Khan 64200, Pakistan; shadab.alam@kfueit.edu.pk (M.S.A.H.); siddiqov@gmail.com (H.-U.-R.S.); furqan.rustam1@gmail.com (F.R.); 2 Department of Computer Science, The University of Bahawalpur, Bahawalpur 63100, Pakistan; muhammad.ibrahim@iub.edu.pk (M.I.); imran.sarwar@iub.edu.pk (I.S.B.); 3 Department of Computer Science, Broward College, Broward County, FL 33301, USA; 4 Department of Information and Communication Engineering, Yeungnam University, Gyeongsan 38541, Korea

**Keywords:** railway track inspection, spectrograms, acoustic signals, machine learning, deep convolution neural networks, LSTM

## Abstract

The periodic inspection of railroad tracks is very important to find structural and geometrical problems that lead to railway accidents. Currently, in Pakistan, rail tracks are inspected by an acoustic-based manual system that requires a railway engineer as a domain expert to differentiate between different rail tracks’ faults, which is cumbersome, laborious, and error-prone. This study proposes the use of traditional acoustic-based systems with deep learning models to increase performance and reduce train accidents. Two convolutional neural networks (CNN) models, convolutional 1D and convolutional 2D, and one recurrent neural network (RNN) model, a long short-term memory (LSTM) model, are used in this regard. Initially, three types of faults are considered, including superelevation, wheel burnt, and normal tracks. Contrary to traditional acoustic-based systems where the spectrogram dataset is generated before the model training, the proposed approach uses on-the-fly feature extraction by generating spectrograms as a deep learning model’s layer. Different lengths of audio samples are used to analyze their performance with each model. Each audio sample of 17 s is split into 3 variations of 1.7, 3.4, and 8.5 s, and all 3 deep learning models are trained and tested against each split time. Various combinations of audio data augmentation are analyzed extensively to investigate models’ performance. The results suggest that the LSTM with 8.5 split time gives the best results with the accuracy of 99.7%, the precision of 99.5%, recall of 99.5%, and F1 score of 99.5%.

## 1. Introduction

The railway network is an important transportation channel that serves as the backbone for many developing countries such as Pakistan. The railway system plays a vital role in a country’s economy by moving people, as well as goods, efficiently and rapidly [1]. With the increase in the number of passengers, the railway network is becoming more sophisticated, burdened, and prone to tear and wear. At the same time, environmental conditions and mechanical forces are speeding up the degradation of railway tracks [2]. Rail tracks are one of the most essential and integral parts of the railway network, and the inspection of rail tracks is essential to prevent accidents and to reduce injuries and casualties [3]. Pakistan is a country where a large number of people use trains for traveling.

In Pakistan, 757 train accidents have been recorded from 2012 to 2017 [4], with an average of 125 accidents per year. Although the ratio of train accidents is higher for developing countries, the United States (US) also has a higher number of accidents. A total of 11,434 rail accidents are reported in the year 2019, causing 7730 injuries and 937 fatalities [5]. A yearly summary of accidents in the US is provided in Table 1. The train accidents in Pakistan and the US show that the lives of hundreds of thousands of people are at risk, and negligence or human error in rail track inspection can increase fatalities and injuries. Proper inspection and timely detection of faults can save countless human lives, as well as reduce the financial losses of the railway network [6]. However, the inspection and maintenance of rail tracks are expensive and time-consuming activities.

For rail track inspection, different non-destructive evaluation (NDE) techniques have been used such as Eddy current testing [8], magnetic flux leakage testing [9], ultrasonic testing [9,10], phased array detection [11], guided wave detection [12], and so on. More details about the tools and techniques used for rail track inspection can be found in [8,13]. In traditional systems, visual inspection and acoustic emission may be included [13]. In the last few years, the Internet of Things (IoT) and machine learning and deep learning networks are gaining large attention for railway track inspection. These approaches are used to develop novel, efficient and effective NDE systems. For this purpose, high-speed cameras [14] and acoustic transducers [15] are installed that provide a blend of traditional inspection methods with the machine learning models. The use of machine learning approaches has eased tasks for human beings with increased processing time and automatic feature extraction. Now, the analysis of long railways tracks is easy and fast when using automated approaches. Even though many techniques are working in rail track inspection, anomaly detection, and classification, numerous challenges remain unsolved, such as a lack of proper datasets, the effective detection of a variety of faults, testing at speeds over 80 km/h, and handling sensors producing big data [13,16].

Before using deep learning, hand-crafted feature engineering was used in applications related to computer vision and audio machine learning. Hand-crafted feature extraction required in-depth, domain-specific knowledge for problem-solving and tuning up the system for better performance [17]. Recently, the automatic feature extraction capability of deep learning has attracted many researchers to work with rail fault detection [18]. Railway track inspection and classification has three main steps. The first step is the preprocessing of the ’wav’ files for eliminating undesired sounds. After preprocessing, feature extraction is performed by using spectrograms. The spectrograms are generated on-the-fly to make the system more flexible. Then, the classification model is trained for railway track fault detection.

**Figure 1:**
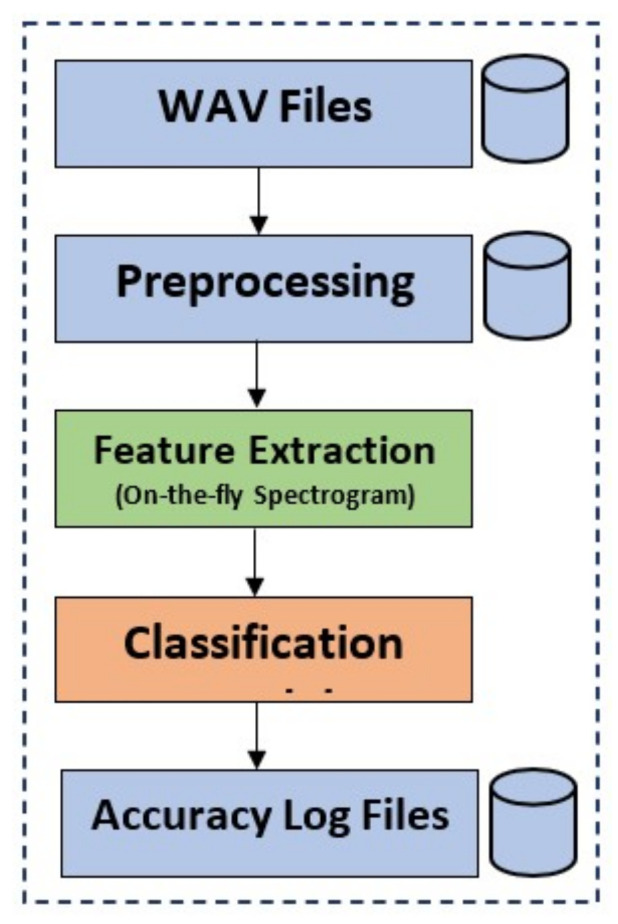
Architecture of the study.

Figure 1 shows steps for providing an understanding of the tasks carried out in this research. In this research, three different deep learning models are tested for their performance. This research focuses on convolutional 1D, convolutional 2D, and long short-term memory (LSTM). The dataset [19] used in this research is a balanced one with three classes including normal, superelevation, and wheel burnt. The first step of preprocessing is to remove the noise of air, rain, and all the dead space in the audio by using a signal envelope function with a threshold of 100 Hz. After cleaning all audio files, they are downsampled to 16 kHz and then split into 1.7 s, 3.4 s, and 8.5 s durations for the experiments. Later, spectrograms are generated on-the-fly. The on-the-fly approach provides flexibility for performing different experiments and increases efficiency at the same time. The key contributions of the study are are follows:

- A novel approach is proposed that can work on the real acoustic dataset for finding railway track faults. The proposed approach uses an on-the-fly approach to generate spectrograms during the training process. It offers flexibility as compared to the traditional approach where the spectrogram dataset is generated before the model training.
- A comparative analysis of the impact of different time slots of the audio sample is carried out. This research also compared the impact of using Mel-Spectrogram and Log-Spectrogram for training purposes.
- Performance analysis is carried out using state-of-the-art techniques with respect to accuracy, precision, recall, and F1 score. In addition, its performance is validated using a statistical *t*-test.

The rest of the paper is organized in the following fashion. Important research works related to the current study are discussed in Section 2. The proposed methodology and related items are provided in Section 3. Section 4 provides the analysis and discussions of the experimental results. In the end, the conclusion is given in Section 5.

## 2. Related Work

Rail track inspections can be generally categorized into two classes: structural inspection and geometric inspection [20]. Structural inspections are conducted to find structural defects which, may include wheel burnt rails or other structural degradation. Geometric inspections are conducted to find geometric irregularities, which may include rail misalignments and other similar problems. Geometric irregularities may also cause structural defects and either of them can lead to train accidents. More information related to structural and geometric defects can be found in [21,22]. A large body of literature can be found that different techniques of shallow learning, deep learning, and transfer learning for rail tracks inspection. In shallow learning, Refs. [23,24,25,26,27] use support vector machine (SVM), Refs. [19,28] use random forest (RF), Ref. [29] uses Adaboost, and [30,31] use principal components analysis (PCA). Regarding the deep learning approach, Refs. [24,32,33,34,35] use convolutional neural networks (CNN), Refs. [36,37,38] use LSTM, and [39] uses a combination of both CNN and RNN called convolutional–recurrent neural network (CRNN). In transfer learning approaches, Refs. [40,41] uses ResNet, and [41] uses a visual geometry group (VGG).

Shafique et al. [19] use tree-based classification models, random forest (RF) and decision tree (DT), which performed well compared to deep learning models for rail track inspection. Jie Liu et al. [23] investigate different variants of SVM, such as twin SVM, LSSVM, etc. They used data from the braking system and tested the model on the KEEL data repository. They focused on the braking system of high-speed trains using an imbalanced dataset and verified their work on publicly available datasets. The sensors are used to collect data related to the braking system. Xavier et al. [24] worked on the dataset collected from 85 miles of railway track. They collected an image dataset from a moving vehicle. They implemented both CNN and SVM techniques in their research. They classify rail fasteners as good, missing, or broken. Study [25] worked to detect geometric defects using the SVM model. The authors used the RAS problem-solving competition 2015 dataset and investigated less severe geometric defects that can develop into more severe geometric defects. Hajizadeh et al. [26] performed a structural inspection to detect structural defects using shallow machine learning techniques. He used SVM and introduced a new metric known as positive and unlabeled learning performance (PULP). The study uses PULP to access the working of classifiers on datasets containing only defective observations.

Along the same lines, Ref. [27] utilize an imbalanced dataset of images taken from the video cameras to detect squats defects. They used a semi-supervised technique to handle vast amounts of unbalanced data. The authors detect structural defects of railway tracks in [28]. The PCA, Kernal-PCA, and histogram match (HM) are used for feature extraction. The extracted features from a dataset comprising non-defective images are utilized to train RF to show the superiority of PCA features for the task at hand. The authors used Adaboost in [29] to detect structural damage and damage specifically related to broken rail fasteners. The study used a Haar-like feature set as the geometrical characteristics of fasteners. Famurewa et al. [30] worked on the geometric aspect of railway tracks. The study detects abnormal patterns in sharp curves by using the PCA algorithm. The experiments are carried out on the Swedish railway network using manually collected data. Lasisi et al. [31] also worked on geometric features of rail by using the PCA algorithm for dimensionality reduction. Kernal-PCA is used along with the SVM to classify four different types of defects in the US Class I railway network.

Dawei Li et al. [32] used a faster region-based CNN for the railway track’s fault. The images are captured using 6 cameras at the speed of a maximum of 15 km/h to detect structural defects of the surface of the metro tunnel. The study suggests that better results can be obtained at a train speed of 5 to 10 km/h. Faghih-Roohi et al. [33] used the CNN model with a manually labeled image dataset collected from rail tracks of the Netherlands. They used three CNN architectures of different sizes and obtained the best result by the deepest architecture. Similarly, Giben et al. [34] suggested a four-layered CNN model on a manually labeled image dataset collected from the US Northeast Corridor for the classification of material used in the rail track. Santur et al. [35] used a 3D laser camera for a more accurate and fast inspection of the rail track. The study used CNN for the binary classification of rail tracks into ’healthy’ or ’faulty’ tracks. The study [36] used an ultrasonic vehicle for finding flaws in the railway track. The LSTM model is used for the detection which shows its feasibility of detection faults at the speed of 15 km/h. Fu et al. [37] worked to detect the structural damage. They used LSTM for classification. They worked on the dataset produced by SIMPACK simulation software.

Bruin et al. [38] used LSTM to detect the presence of trains on the track. They worked to detect different structural faults such as ballast degradation, insulated joint defect, conductive object, etc. Qin et al. [39] used a hybrid neural network with the data generated in SIM-PACK simulation software. Four classes are used for experiments, including normal, LD fault, AS fault, and AD fault. Chen et al. [41] worked on image data randomly selected from inspection videos. They took the images of current-carry rings and manually labeled them as ’normal’ or ’faulted’. They used VGG, ResNet50, and Res2Net50 models for performance evaluation. They trained and tested the models to predict normal and defective current carry rings. Yang et al. [40] analyzed the structural defects, e.g., rail end better, localized surface collapse, and turning points, etc. They used ResNet and fully CNN (FCN) for the problem. The experimental results show that ResNet is slightly better than FCN with 0.77 and 0.76 precision scores for ResNet and FCN, respectively. Mahfuz et al. [42] worked on a dummy system using an Ultrasonic sensor and Arduino Mega. They worked to detect cracks on railway tracks without using machine learning or deep learning models. The study [38] worked to find faults on railway track circuits. They used LSTM to determine different faults in circuits such as conductive objects, electrical disturbance, etc.

## 3. Materials and Methods

**Figure 2:**
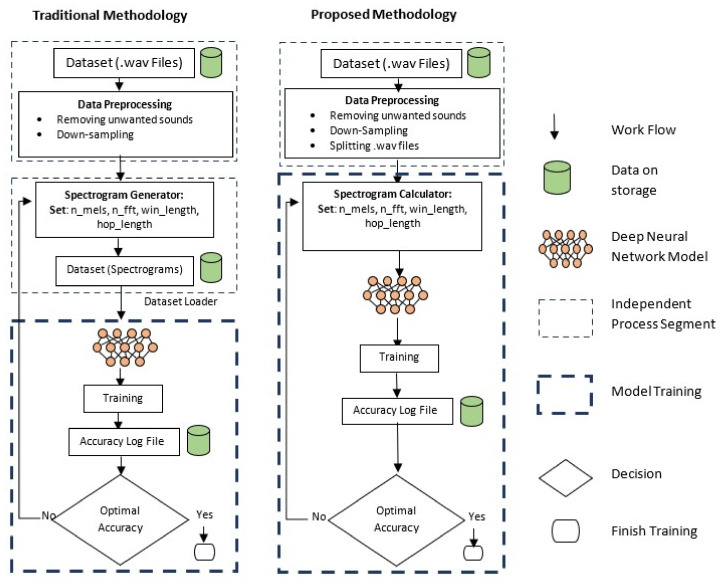
Proposed methodology in contrast with traditional acoustic-based research methodology.

The architecture of the proposed approach is given in Figure 2. The main advantage of this research is generating the spectrograms at a run time that makes the system more flexible than the traditional approaches. Traditionally, spectrograms are generated and stored on a disk before feeding them into the machine and deep learning networks, which makes the network slower and requires more space. For example, MusicNet [43], a large labeled dataset publicly available to learn music features requires a substantial disk size. This dataset is 20 GB in size, and creating different types of spectrograms using different parameters, it can take up to 1TB of disk space.

Even if the aspect of high storage requirement is ignored, sound to spectrograms conversion requires a large amount of time. For the experiments, this study uses Librosa, a famous Python library for converting sound to spectrograms. On Google Colab, it took about 20 h and 48 min for converting sound to spectrogram for 1240 samples, and it took about 29 h and 40 min for converting 1850 sound to spectrogram conversion on PC. Librosa takes 1.0 min and 0.96 min on average for creating one spectrogram on both CPU and GPU, respectively. Optimizing the parameters for creating better spectrograms to obtain more accurate results can consume even larger time. So, in this research, instead of generating spectrograms as a separate and independent process, they are calculated on-the-fly as a layer of the neural network using Kapre [44].

### 3.1. Dataset

**Figure 3:**
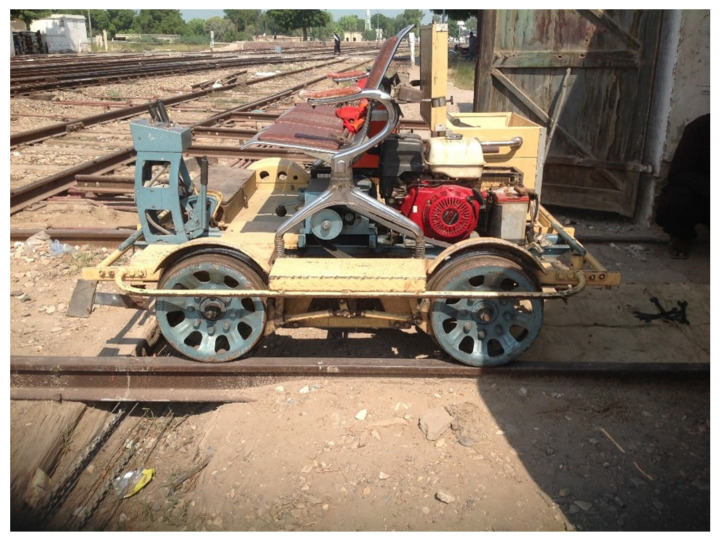
The railway cart equipped with microphones for the data collection [19].

The dataset used in this research is collected by [19]. It has 720 mono channel audio ’wav’ samples and is collected in the area of Sadiq Abad Junction, Pakistan. Figure 3 shows the setup used for the data collection. An onsite setup is used where 2 microphones were mounted at a distance of 1.75 inches from wheel and track contact point. A mechanical cart is used for data collection at an average speed of 35 km per hour. Two ECM-X7BMP Unidirectional electric condensers are used, which are supplied with a 3-pole locking mini plug. The sensitivity of the microphone is −44.0 ± 3 dB, while the output impedance is 1.2 kΩ ± 30%. The dynamic range is 88 dB, the signal-to-noise ratio is 62 dB, and the operating voltage is 5.0 V. The microphones are connected through a wire and are unidirectional. For further details about the data collection process, the readers are referred to [19].

The dataset has three classes, i.e., normal, superelevation, and wheel burnt. It is a balanced dataset with each class containing 240 samples with a length of 17 s each. For experiments, every 17 s, the audio file is split into 1.7 s, 3.4 s, and 8.5 s time intervals (see Table 2).

### 3.2. Data Preprocessing

**Figure 4:**
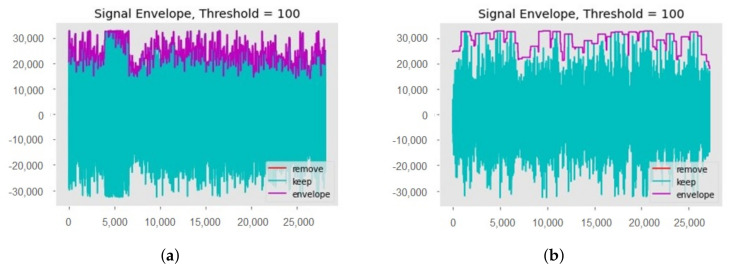
Signal envelope function: (**a**) Signal envelope plot; (**b**) Zoomed portion of the plot.

Data preprocessing is one of the most important steps required for machine learning models, as the learning process is affected by the preprocessing. For preprocessing, first of all, unwanted sounds are removed from all collected audio samples. These unwanted sounds include the sound of rain and wind, etc. Then, dead space from the audio is removed by using a threshold value, see Figure 4. A threshold value of 100 Hz is used to clean the audio samples, so any frequency below 100 Hz will be removed. Finally, the down-sampling technique is used at the rate of 16,000 [45]. This down-sampling is used during the splitting of all audio files for specific time intervals and saved back to the storage. For the research purpose, audio is split into 1.7 s, 3.4 s, and 8.5 s. Figure 4b, is the zoomed portion of the plot Figure 4a.

### 3.3. Data Augmentation

Undoubtedly an appropriate artificial neural network (ANN) model and its tuned-up hyperparameters are essential factors for the development of a good model; however, appropriate data are equally important for testing and proving the efficacy of the ANN model.

**Figure 5:**
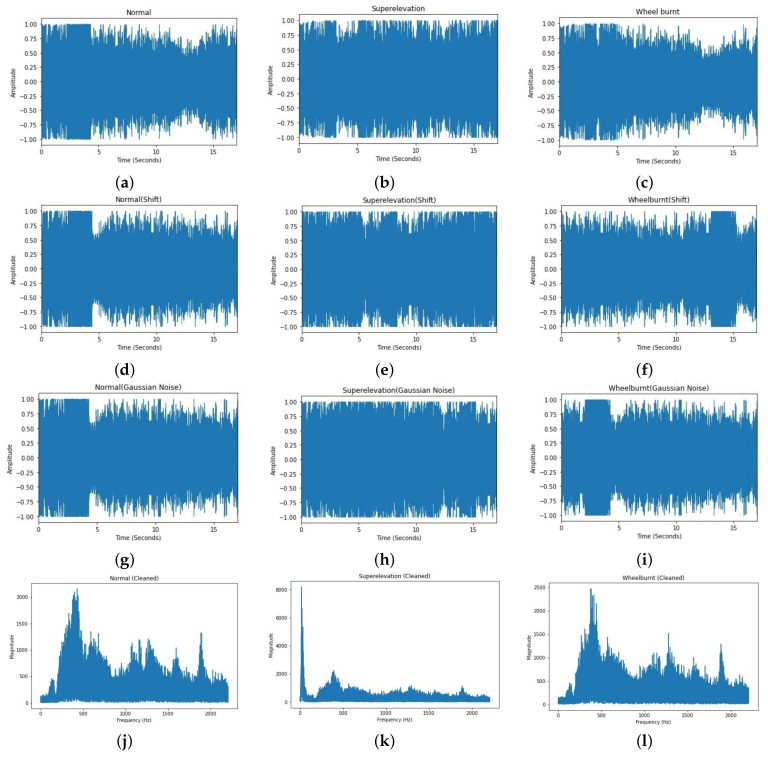
Time domain representation of different classes: (**a**) Normal class, (**b**) Superelevation class, and (**c**) Wheel burnt class; Time domain representation with shift augmentation: (**d**) Normal class, (**e**) Superelevation class, and (**f**) Wheel burnt class; Time domain representation with Gaussian noise augmentation: (**g**) Normal class, (**h**) Superelevation class, and (**i**) Wheel burnt class; Frequency representation of different classes, cleaned using signal envelope function and without augmentation: (**j**) Normal class, (**k**) Superelevation class, and (**l**) Wheel burnt class.

If the dataset is inappropriate and small, even a good model’s result will not be acceptable. So, to enhance the suitability of the data, different audio data augmentation or audio deformation techniques are applied without disturbing the semantic labeling of data, and these techniques are discussed as follows. The study uses audiomentations [46], a Python library for augmentation. Audio augmentations used in this research are as follows:

- **Shift**: In this technique, audio samples are shifted backward or forward. This shifting can be performed with or without rollover. Rollover is of Boolean type; if it is set to true, then the samples rolled beyond the last or the first position are re-injected into the audio. Parameters used for shift are min_fraction = −0.7, max_fraction = 0.7, rollover = ’True’, *p* = 0.7. Implementation of shift can be seen in Figure 5d–f.
- **Gaussian Noise**: In this technique of audio data deformation, Gaussian noise is added into the raw audio before feeding it into the ANN. Parameters which we used to add Gaussian noise are min_aplitude = 0.003, max_amplitude = 0.022, and *p* = 0.7. Implementation of Gaussian noise can be seen in Figure 5g–i.

### 3.4. Proposed Methodology

This research uses three deep learning models for experiments including Convolutional 1D (Conv1D), Convolutional 2D (Conv2D), and LSTM, which belong to the RNN family [47]. RNN and LSTM have proved to be better models as compared to the hidden Markov Model in different domains, e.g., hand-writing recognition [48], emotion recognition [49], generating music composition [50], and speech recognition [51], etc. LSTM consists of a connected memory cell to form an artificial neural network. Each cell has three gates, an input gate, output gate, and forget gate, that are used to perform the read function, write function, and reset function, respectively. Precisely, the input of the input cell is multiplied by the input gate. The output of the output cell is multiplied by the output gate, and previous cell values are multiplied by the forget gate. These functions can be defined by the equations as follows:

(1) $$\begin{array}{c} i^{t}=\sigma \left(W_{xi}x^{t}+W_{hi}h^{t-1}+W_{ci}c^{t-1}+b_{i}\right) \end{array}$$

(2) $$\begin{array}{c} f^{t}=\sigma \left(W_{xf}x^{t}+W_{hf}h^{t-1}+W_{cf}c^{t-1}+b_{f}\right) \end{array}$$

(3) $$\begin{array}{c} a^{t}=tanh\left(W_{xc}x^{t}+W_{hc}h^{t-1}+b_{c}\right) \end{array}$$

(4) $$\begin{array}{c} c^{t}=f^{t}⊙c^{t-1}+i^{t}⊙a^{t} \end{array}$$

(5) $$\begin{array}{c} o^{t}=\sigma \left(W_{xo}x^{t}+W_{ho}h^{t-1}+W_{co}c^{t}+b_{o}\right) \end{array}$$

(6) $$\begin{array}{c} h^{t}=o^{t}⊙tanh\left(c^{t}\right) \end{array}$$

where *i* is the input gate, *f* is the forget gate, *o* is the output gate, *a* is the cell input activation vector, *c* is a self-connected state vector, and all have a size similar to *h* which is the hidden vector. $W_{ci}$, $W_{co}$, and $W_{cf}$ are all peephole connection weights which are diagonal. So, element *m* in each gate vector only receives input from element *m* of the cell vector.

In this research, bidirectional LSTM (BiLSTM) is used, which increases predictions accuracy as compared to simple LSTM [52]. BiLSTM uses two independent LSTM networks: one network learns data from start to end, and the second network learns data from end to start. The following are the building blocks of our proposed state-of-the-art model.

#### 3.4.1. STFT Layer

During the audio recording analog signals are converted into digital signals and the number of samples, recorded during one second, becomes the sampling rate. To convert the time-domain signal into a frequency-domain, Discrete Fourier Transform (DFT) is used [53], which can be represented as:

(7) $$S\left[k\right]=\sum_{n=0}^{N-1}x\left[n\right]e^{-j2\pi kn/N}$$

where X[k] is the frequency-domain output, x[n] is the *n*th sample in the time-domain audio input, *N* is the length of the window, *n* is the discrete time index, *k* is the discrete frequency index. For real-valued inputs, the frequency domain output X[k] for k∈[1,N/2] is equal to the output X[k] for k∈[N/2,N−1] in reverse order.

Short-Time Fourier Transform (STFT) is a sliding window concept of DFT. Instead of converting the whole time-domain signal into a frequency domain signal, we cut the signal into a small window and then perform transformation [54]. It is the standard way to perform audio analysis-based applications. These segmented signals can be represented as:

(8) $$x_{l}=w\left[n\right]x\left[n+lL\right],0\leq n\leq N-1$$

where *l* is the index of the frame, *N* is the length of the window, *L* is the hop size, and *n* is index to local time. The parameters used for the model are (n_fft=512, win_length = 400, hop_length = 160), and the Hann Window function is used:

(9) $$w\left(k\right)=0.5\left(1-cos\left(\frac{2\pi k}{k-1}\right)\right),\;k=1,2,\ldots ,K$$

**Figure 6:**
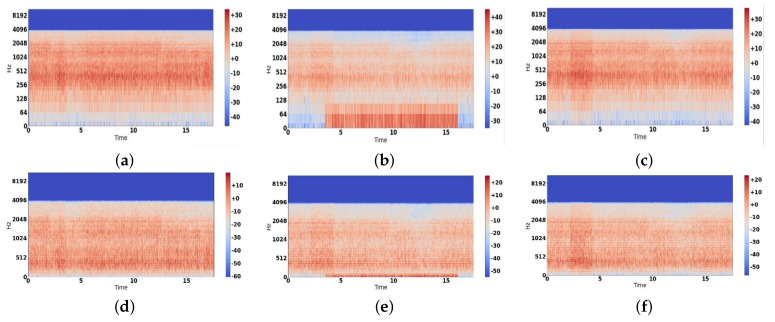
Log spectrogram representation of different classes: (**a**) Normal class, (**b**) Superelevation class, and (**c**) Wheel burnt class; Mel spectrograms after applying Mel filter bank: (**d**) Normal class, (**e**) Superelevation class, and (**f**) Wheel burnt class.

By stacking up the STFTs, a spectrogram is formed, which is the representation of time-frequency intensity [55]. Fast Fourier transform (FFT) is an algorithm that is used as a quick resource for computing STFT in digital computers [56,57]. We usually obtain the log-spectrogram, which is not from a human perception, (see Figure 6a) because the human ear is more sensitive to low frequencies as compared to high frequencies. To convert log-spectrograms from a human perception, Stevens et al. introduced the Mel frequency scale in 1937 [58]. It was an attempt to quantify the pitch in a way that reflects the same differences between Mel-scale pitch and perceived pitch, irrespective of the frequency in Hertz [58]. Stevens et al. [59] and W. Koening [60] also tried to modify the original Mel-scale, so there is no single formula available in literature [61]. One of the formulas mentioned by O’Shaughnessy in his book [62] is given in Equation (10).

(10) $$m=2595log_{10}\left(1+\frac{f}{700}\right)$$

**Figure 7:**
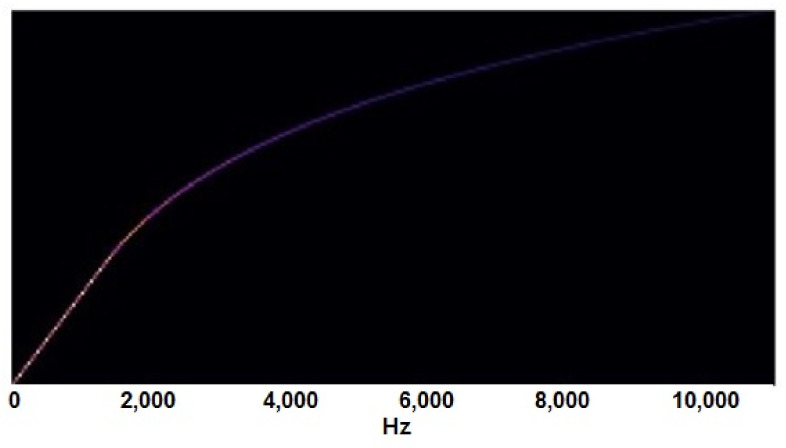
Mel filter bank used for creating Mel-spectrograms.

After obtaining the Mel scale conversion, Mel filter banks can be constructed, which are then multiplied with previously obtained spectrograms to obtain Mel-scale spectrograms [63]. The obtained Mel filter bank can be seen in Figure 7, and from this Mel filter bank, we obtain Mel-spectrograms as given in Figure 6.

#### 3.4.2. Batch Normalization Layer

A batch normalization layer is used in the start to normalize data before feeding them into the network [64]. Equation (11) normalizes the data by using a standard normal distribution with zero mean and one variance. This normalization is necessary because the activation function which is used at the first layer of all three models, Conv1D, Conv2D, and BiLSTM, is a hyperbolic tangent:

(11) $$\alpha_{i}^{norm}=\Upsilon_{i}\frac{\alpha_{i}-\mu }{\delta_{i}}+\beta_{i}$$

where $\alpha_{i}$ is the actual activation function of a specific neuron, μ is the mean value, $\delta_{i}$ is the standard deviation of the inputting neuron, $\Upsilon_{i}$ is the expansion factor, $\beta_{i}$ is the translation factor, and $\alpha_{i}^{norm}$ is the new normalized value.

#### 3.4.3. Maximum (Max) Pooling Layer

The max pooling layer is used after every convolutional layer in the Conv1D and Conv2D models and the LSTM model to reduce the number of parameters. The total trainable parameters without the max-pooling layer were 131,092,339, and with yjr max-pooling layer, they were reduced to 544,627 in the Conv2D model.

#### 3.4.4. Dense Layer

The dense layer consists of neurons, and the inputs of these neurons are associated weights; after performing some linear functions, they pass outputs to the next layer [65]. All the neurons of a dense layer are connected to the input and output layers. A dense layer can be presented as:

(12) X=f(Y×w+b)

where *Y* is the input layer, *w* is the weight, *b* is th ebias vector, *f* is the activation function, and *X* is the output layer [66].

#### 3.4.5. Flatten Layer

The flatten layer is used to convert data into a 1D array, which is the output of convolutional layers. Finally, the output of the flatten layer is connected to a fully connected layer, which eventually performs the final classification.

#### 3.4.6. Drop Out Layer

Overfitting is a severe issue in the field of machine learning and deep learning. Overfitting is a situation in which the model gives satisfying results during the training process, but in the testing process, it gives unsatisfactory results. Normally, it happens when multiple neurons detect the same results repeatedly [67] and neurons have to be dropped. The drop out layer is presented as follows:

(13) $$z_{i}^{\left(l+1\right)}=w_{i}^{\left(l+1\right)}y^{l}+b_{i}^{\left(l+1\right)}$$

Consider a network of *L* hidden layers, where $l\in 1,\ldots ,L,z^{\left(l\right)}$ denotes the vector of inputs into layer *l*, $y^{\left(l\right)}$ denotes the vector of outputs from layer *l*, $\left(y^{\left(0\right)}=x\right)$ is the input, and $w^{\left(l\right)}$ and $b^{\left(l\right)}$ are the weights and biases, respectively, at layer *l*.

#### 3.4.7. Softmax Layer

Softmax is the last layer of the deep learning model. It is used for multiclass classification in which the output is characterized categorically [67]. It is an activation function and very much important in ANN. Moreover, it is used to decide whether neurons are active or otherwise. The primary objective of the Softmax layer is to highlight the maximum value in the neurons. It assigns one as the maximum weight of neurons and sets other neurons’ weight to zero. The Softmax function can be presented as follows:

(14) $$S\left(y_{i}\right)=\frac{e^{yi}}{\sum_{k}e^{y^{k}}},\;k=1,2,\ldots ,k$$

where *y* is the input layer, and *S* is the output layer.

## 4. Results and Discussion

For experiments, this study uses the Intel Core i3-4010U@1.70 GHz CPU, with 8 GB RAM and a GTX 550 graphics card. Python 3.8 is selected as the development environment. Several detailed experiments are performed to find the best-performing deep learning model to classify the rail track into three different types, i.e., normal, wheel burnt, and superelevation. For experiments, three deep learning models, Conv1D, Conv2D, and LSTM, are used and all these models are trained and tested for three different lengths of audio samples. Detailed scenarios and their performances are compared as below:

- **Scenario 1**—each audio sample is split into 1.7 s, and Conv1D, Conv2D, and LSTM are trained and tested against all three classes.
- **Scenario 2**—each audio sample is split into 3.4 s, and Conv1D, Conv2D, and LSTM are trained and tested against all three classes.
- **Scenario 3**—each audio sample is split into 8.5 s, and Conv1D, Conv2D, and LSTM are trained and tested against all three classes.

Audio data augmentation is applied to the best-performing model. Augmentation is performed on both datasets, first on the training dataset, and then on testing data to validate the model’s generalization. The basic reason for splitting audio into smaller chunks is not to overload the neural network model during training and to avoid overfitting.

### 4.1. Results for Scenario 1

By splitting a 17 s audio sample into 1.7 s, we have 10 files for each sample. A total of 5100 files are used for training in the first scenario. After performing training, the performance and training time of all three models can be observed in Table 3. All models are trained for 30 epochs and 10-fold cross-validation. In this scenario, Conv2D performed better than the other two models, but it took a significantly longer time than the Conv1D and LSTM models. LSTM took the least time for training.

### 4.2. Results for Scenario 2

For the second scenario, all 17 s audio samples are split into 3.4 s for the training purpose. In this scenario, we have 2550 files for training our deep learning models. In this split time, LSTM proves to be the most efficient model again, and Conv1D proves the most effective model, with 95% accuracy as shown in Table 4.

### 4.3. Results for Scenario 3

In this scenario, audio samples are split into 8.5 s in length. This split gives maximum accuracy among all three time-split experiments. LSTM performed better than Conv1D in terms of all performance evaluation parameters and almost equally well as Conv2D, but LSTM proved to be the most efficient model by taking the least time for training, as shown in Table 5.

So, among all three scenarios, the combination of 8.5 s split time with LSTM proves the best combination, as LSTM gives 99% accuracy, which is equivalent to the accuracy of the Conv2D model, but LSTM took about half the training time as compared to Conv2D, as shown in Table 5 and Table 6 shown the results with each augmentation approach.

Now, to deeply examine LSTM’s effectiveness and efficiency, we performed data augmentation. We performed audio augmentation of the following types:

(**a**) Gaussian noise;
(**b**) Shift;

which enhanced the test dataset from 210 to 630 files. Table 7 and Table 8 showing the confusion matrix values with proposed approaches.

To prove the effectiveness of our LSTM model, we performed testing on the augmented dataset using the model which was previously trained on an un-augmented training dataset. In this case, as the training was performed on an un-augmented dataset, our model made 63 wrong predictions out of 630, as shown in Table 9 and Table 10 shown 17 wrong predictions using augmented training dataset and augmented test dataset.

### 4.4. Discussion

In this section, a detailed analysis of experiments is presented. Three deep learning models, Conv1D, Conv2D, and LSTM, have been thoroughly experimented with. Deep learning models allow the flexibility needed for creating an on-the-fly spectrogram as a layer of the neural network, which is not available in the shallow machine learning models. For experiments with shallow learning, first, an offline dataset of spectrograms must be created. Creating an offline dataset of spectrograms is time-consuming and requires large storage. For the current research, the estimated time required for creating spectrograms can be seen in Table 11, and tuning the parameters of spectrograms requires a large time. In Table 11, it can be observed that for all the cases, the time required for the generation of the Spectrogram dataset is much higher than the training time required by each model, except for the case of 8.5 s, where the Conv 2D’s training time is very close to spectrogram generation time. However, due to the slowness of conv2D, it was dropped, and we selected the LSTM model instead.

**Figure 8:**
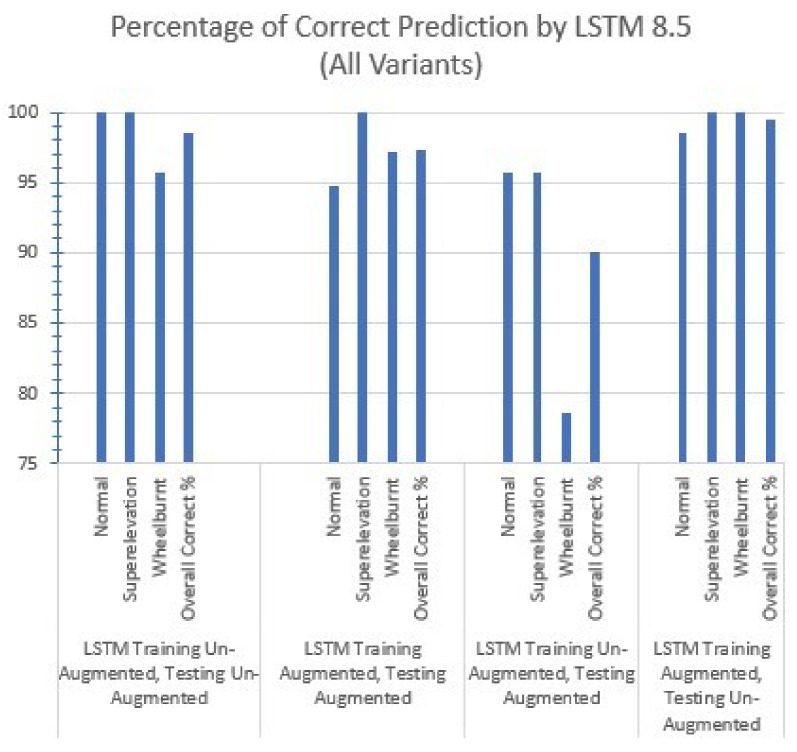
Percentage of correct predictions made by all variants of LSTM with 8.5 s split time.

It is, therefore, better to utilize deep learning models’ flexibility to create on-the-fly spectrograms and feed them directly into models instead of creating them as a separate and independent process. Three deep learning models, i.e., Conv1D, Conv2D, and LSTM, are incorporated in this research, and each model is trained and tested for three different time splits of the audio dataset, i.e., 1.7 s, 3.4 s, and 8.5 s. LSTM gives the best accuracy using the 8.5 s split time with the least training time. The LSTM model consists of an LSTM layer, a dense layer, a dropout layer, and a softmax layer. LSTM memory cells remember the past data, and it is used for time-series analysis. The percentage of correct predictions made by different variants of LSTM can be seen in Figure 8.

Table 12, Table 13 and Table 14 show the architectural details of Conv1D, Conv2D, and LSTM models used for experiments.

### 4.5. Results Using Log Spectrogram

**Figure 9:**
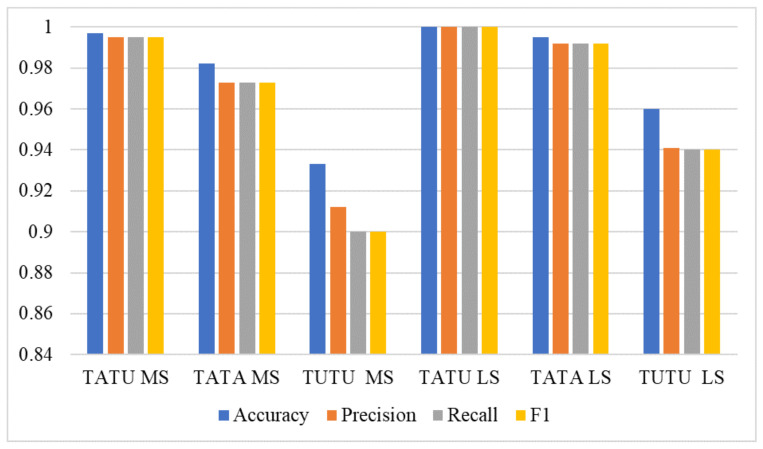
LSTM comparison using log spectrogram and Mel spectrogram.

Experiments are performed using the log spectrogram and the LSTM model with an 8.5-second sub-sample. The performance of the LSTM model is significant with log spectrogram as compared to the Mel spectrogram, as it achieves the highest accuracy 100% with log spectrogram when we used augmentation on training but not on test data. Overall, the performance with the log spectrogram is better as compared to the Mel spectrogram in terms of all evaluation parameters, as shown in Table 15. Figure 9 shows the comparison between the Mel spectrogram (MS) and log spectrogram (LS), while TATU, TATA, and TUTA indicate training augmented, testing un-augmented; training augmented, testing augmented; and training un-augmented, testing augmented, respectively. Log spectrogram took 5.08 h of training, which is less than the Mel-scale spectrograms, which took 7.5 h of training.

We also deploy state-of-the-art deep learning models in comparison with the proposed approach for a fair comparison. We deploy three pre-trained models including VVG16, InceptionV3, and ResNet-50. The results given in Table 16 indicate that other deep learning approaches do not provide high performance similar to the proposed approach. These models do not provide better results with our used dataset without an on-the-fly approach. Apparently, the reason is the complex architecture of the models that need large datasets to produce better results. The current dataset being with a small feature set is not able to provide high accuracy with these models.

### 4.6. Statistical Significance Test

We carried out the statistical significance test to show the significance of the proposed approach LSTM with log spectrogram [68,69]. We deployed the T-test on the results of LSTM with both the Mel spectrogram and the log spectrogram. In the output of the *t*-test, we have null hypotheses and alternative hypotheses as follows:

- Null hypothesis ($H_{o}$): There is no significant difference between the result of LSTM using log spectrogram and LSTM using the Mel spectrogram.
- Alternative hypothesis ($H_{a}$): There is a significant difference between the result of LSTM using log spectrogram and LSTM using the Mel spectrogram.

If the *t*-test accepts the null hypothesis, it means there is no significant difference between the result of LSTM using a log spectrogram and LSTM using a Mel spectrogram and vice versa. The *t*-test rejects the null hypothesis and accepts the alternative hypothesis because the t statistic value is greater than the critical value. The t statistic = 4.889 and critical value = 0.540 on the results of LSTM. This *t*-test result shows that LSTM with log spectrogram achieves better results, which are statistically significant.

### 4.7. Significance of Using Automated Approach for Railway Track Inspection

The proposed approach has several advantages over the current manual inspection system in Pakistan.

- Railway tracks are vulnerable to damage and deterioration by several extreme events, such as buckling by heat [70]. Extreme heat in Pakistan leads to buckling, causing severe accidents. The manual railway track inspection system in Pakistan is unable to perform efficient and effective inspections, which can be augmented by the current proposed automated system.
- Every year, Pakistan faces flooding, which may cause cutting slope failures [71]. Additionally, ballasts may be washed away [72]. With an automated system, the fast and accurate detection of such defects is possible.
- Of the several sensors and methods used for railway track fault detection, such as cameras, radiography, thermal sensors, and optical-laser-based sensors, a microphone can provide fault detection at a higher speed [73]. Despite its shortcoming of being affected by noise, it is still possible to find surface defects, wheel defects, etc., using a microphone setup.
- This study used an on-the-fly approach, which is appropriate and effective as compared to previous studies on train track fault detection.
- This study outperforms the accuracy of previous study [19] in terms of accuracy. This study also takes the advantage of on-the-fly extraction of spectrograms, without saving spectrogram dataset on the disk. On-the-fly approach makes the system flexible, so we can perform experiments with different lengths of audio with different types of spectrograms such as the Mel-Spectrogram and the Log-Spectrogram.

We believe that these features make the current approach suitable enough to be utilized for real-world railway tracks inspection in Pakistan.

## 5. Conclusions

Keeping in view the importance of railway track inspection for fault detection in saving human lives, this study presents an automatic railway inspection approach using audio data with a novel deep learning LSTM model. In addition, Conv1D and Conv2D models are also tested for the same task. Each sample of 17 s is split into subsamples of 1.7 s, 3.4 s, and 8.5 s to reduce the processing time and computational complexity. Three deep learning models, Conv1D, Conv2D, and LSTM, are extensively studied for each variation of split time. Mel spectrograms and log spectrograms are used for feature extraction and spectrograms are generated on-the-fly as a layer of the deep learning model. This research provides a flexible approach compared to the traditional approach in which the audio dataset is converted into spectrograms’ dataset and stored prior to models’ training that requires substantial time and space. Several experiments are performed for in-depth investigation of models’ performance where, firstly, the un-augmented dataset is used in several experiments with unique combinations of each model with each split time. Secondly, LSTM with 8.5 s split time proved to be the best performer, and it is further tested with augmented training and augmented testing datasets. Finally, after performing augmentation on both training and testing datasets, experiments are performed with 2850 and 630 samples for training and testing, respectively. The results show that LSTM provides an accuracy of 98.2%, a precision of 97.3%, a recall of 97.3%, and an F1 score of 97.3%. The model trained on the augmented dataset obtains an accuracy of 99.7% against the un-augmented test dataset.

## Figures and Tables

**Table 1:** Railway accidents stats in Pakistan [7].

Year	No. of Deaths	No. of Injured
2015	40	No Reports
2016	28	150
2017	16	10
2018	No Reports	32
2019	97	100
2020	19	No Reports
2021	32	64

**Table 2:** Number of samples in each time split, with and without augmentation.

Audio Length	1.7 s	3.4 s	8.5 s
Number of samples without augmentations	5800	2830	1160
Number of samples with augmentations	N/A	N/A	3480

**Table 3:** Performance evaluation and training time required by all three models with 1.7 s split time.

Model	Accuracy	Precision	Recall	F1	Training (Hours)
Conv1D	0.949	0.932	0.923	0.924	8.66
Conv2D	0.965	0.955	0.947	0.948	16.9
LSTM	0.933	0.911	0.899	0.900	6.80

**Table 4:** Performance evaluation and training time required by all three models with 3.4 s split time.

Model	Accuracy	Precision	Recall	F1	Training (Hours)
Conv1D	0.959	0.939	0.938	0.938	7.5
Conv2D	0.952	0.930	0.929	0.928	15.08
LSTM	0.930	0.920	0.895	0.893	6.75

**Table 5:** Performance evaluation and training time required by all three models with 8.5 s split time.

Model	Accuracy	Precision	Recall	F1	Training (Hours)
Conv1D	0.972	0.958	0.959	0.958	7.91
Conv2D	0.990	0.985	0.986	0.986	16.25
LSTM	0.990	0.986	0.986	0.986	7.5

**Table 6:** Performance evaluation using all variants of LSTM with 8.5 s split time.

LSTM Model	Accuracy	Precision	Recall	F1
Training augmented, Testing un-augmented	0.997	0.995	0.995	0.995
Training augmented, Testing augmented	0.982	0.973	0.973	0.973
Training un-augmented, Testing augmented	0.933	0.912	0.90	0.900

**Table 7:** Confusion matrix of LSTM 8.5 s split, un-augmented training dataset and un-augmented test dataset.

Actual class	Normal	70	0	0
	Superelevation	0	70	0
	Wheel burnt	3	0	67
	Predicted class	Normal	Superelevation	Wheel burnt

**Table 8:** Confusion matrix of LSTM 8.5 s split, augmented training dataset and un-augmented test dataset.

Actual class	Normal	69	0	1
	Superelevation	0	70	0
	Wheel burnt	0	0	70
	Predicted class	Normal	Superelevation	Wheel burnt

**Table 9:** Confusion matrix of LSTM 8.5 s split, un-augmented training dataset and augmented test dataset.

Actual class	Normal	201	0	9
	Superelevation	9	201	0
	Wheel burnt	45	0	165
	Predicted class	Normal	Superelevation	Wheel burnt

**Table 10:** Confusion matrix of LSTM 8.5 s split, augmented training dataset and augmented test dataset.

Actual class	Normal	199	0	11
	Superelevation	0	210	0
	Wheel burnt	6	0	204
	Predicted class	Normal	Superelevation	Wheel burnt

**Table 11:** Time required for signal envelop function.

Split Time	Time Required to Generate Spectrogram Dataset (Hours)	Training Time for Conv 1D (Hours)	Training Time for Conv 2D (Hours)	Training Time for LSTM (Hours)
1.7 s	85	8.66	16.9	6.8
3.4 s	42.5	7.5	15.08	6.75
8.5 s	16.66	7.91	16.25	7.5

**Table 12:** Layers detail of Conv1D model.

Layer	Values	Output Shape
get_melspectrogram_layer	n_mels = 128, n_fft = 512, win_length = 400, hop_length = 160, sample_rate = 16,000, return_decibel = True	(None, 500, 257, 1)
filterbank	n_mels = 128, n_fft = 512, win_length = 400, hop_length = 160	(None, 500, 128, 1)
batchNormalization	Axis = 2	(None, 500, 128, 1)
Conv1D	Activation = Hyperbolic tangent, Filters = 8, Kernel = 4	(None, 500, 125, 8)
maxPooling2D	2 × 2	(None, 250, 62, 8)
Conv1D	Activation = ReLU, Filters = 16, Kernel = 4	(None, 250, 59, 16)
maxPooling2D	2 × 2	(None, 125, 29, 16)
Conv1D	Activation = ReLU, Filters = 32, Kernel = 4	(None, 125, 26, 32)
maxPooling2D	2 × 2	(None, 62, 13, 32)
Conv1D	Activation = ReLU, Filters = 64, Kernel = 4	(None, 62, 10, 64)
maxPooling2D	2 × 2	(None, 31, 05, 64)
Conv1D	Activation = ReLU, Filters = 128, Kernel = 4	(None, 31, 02, 128)
globalMaxPooling2D	-	(None, 128)
Dropout	Rate = 0.1	(None, 128)
Dense	Activation = ReLU	(None, 64)
Dense	Activation = Softmax	(None, 3)

**Table 13:** Layers detail of Conv2D model.

Layer	Values	Output Shape
get_melspectrogram_layer	n_mels = 128, n_fft = 512, win_length = 400, hop_length = 160, sample_rate = 16,000, return_decibel = True	(None, 500, 257, 1)
filterbank	n_mels = 128, n_fft = 512, win_length = 400, hop_length = 160	(None, 500, 128, 1)
batchNormalization	Axis = 2	(None, 500, 128, 1)
Conv2D	Activation = Hyperbolic tangent, Filters = 8, Kernel = 7 × 7	(None, 500, 128, 8)
MaxPooling2D	2 × 2	(None, 250, 64, 8)
Conv2D	Activation = ReLU, Filters = 16, Kernel = 5 × 5	(None, 250, 64, 16)
MaxPooling2D	2 × 2	(None, 125, 32, 16)
Conv2D	Activation = ReLU, Filters = 16, Kernel = 5 × 5	(None, 125, 32, 16)
MaxPooling2D	2 × 2	(None, 63, 16, 16)
Conv2D	Activation = ReLU, Filters = 32, Kernel = 3 × 3	(None, 63, 16, 32)
MaxPooling2D	2 × 2	(None, 32, 08, 32)
Conv2D	Activation = ReLU, Filters = 32, Kernel = 3 × 3	(None, 32, 08, 32)
Flatten	-	(None, 8192)
Dropout	Rate = 0.2	(None, 8192)
Dense	Activation = ReLU	(None, 64)
Dense	Activation = Softmax	(None, 3)

**Table 14:** Layers detail of LSTM model.

Layer	Values	Output Shape
get_melspectrogram_layer	n_mels = 128, n_fft = 512, win_length = 400, hop_length = 160, sample_rate = 16,000, return_decibel = True	(None, 500, 257, 1)
filterbank	n_mels = 128, n_fft = 512, win_length = 400, hop_length = 160	(None, 500, 128, 1)
batchNormalization	Axis = 2	(None, 500, 128, 1)
Reshape	Value = −1	(None, 500, 128)
Dense	Activation = Hyperbolic tangent	(None, 500, 64)
BiLSTM	Units = 64, return_sequences = True	(None, 500, 64)
Concatenate (Skip connection)	Axis = 2	(None, 500, 128)
Dense	Activation = ReLU	(None, 500, 64)
MaxPooling1D	-	(None, 250, 64)
Dense	Activation = ReLU	(None, 250, 32)
Flatten	-	(None, 8000)
Dropout	Rate = 0.2	(None, 8000)
Dense	Activation = ReLU	(None, 32)
Dense	Activation = Softmax	(None, 3)

**Table 15:** Results of LSTM model with log Spectrogram.

LSTM Model (8.5 s)	Accuracy	Precision	Recall	F1
Training augmented testing un-augmented (Log Spectrogram)	1	1	1	1
Training augmented testing augmented (Log Spectrogram)	0.995	0.992	0.992	0.992
Training un-augmented testing augmented (Log Spectrogram)	0.96	0.941	0.94	0.94

**Table 16:** Results using pre-trained deep learning models on the original dataset.

Model	Accuracy	Precision	Recall	F1 Score
VVG-16	0.41	0.41	0.51	0.45
InceptionV3	0.35	0.35	0.35	0.35
ResNet-50	0.42	0.42	0.42	0.42

## Data Availability

Not applicable.
